# Cost-effectiveness of using artificial intelligence versus polygenic risk score to guide breast cancer screening

**DOI:** 10.1186/s12885-022-09613-1

**Published:** 2022-05-06

**Authors:** Shweta Mital, Hai V. Nguyen

**Affiliations:** School of Pharmacy, Memorial University of Newfoundland, 300 Prince Philip Drive, St. John’s, NL A1B 3V6 Canada

**Keywords:** Artificial intelligence, Polygenic risk scores, Breast cancer screening, Cost-effectiveness

## Abstract

**Background:**

Current guidelines for mammography screening for breast cancer vary across agencies, especially for women aged 40–49. Using artificial Intelligence (AI) to read mammography images has been shown to predict breast cancer risk with higher accuracy than alternative approaches including polygenic risk scores (PRS), raising the question whether AI-based screening is more cost-effective than screening based on PRS or existing guidelines. This study provides the first evidence to shed light on this important question.

**Methods:**

This study is a model-based economic evaluation. We used a hybrid decision tree/microsimulation model to compare the cost-effectiveness of eight strategies of mammography screening for women aged 40–49 (screening beyond age 50 follows existing guidelines). Six of these strategies were defined by combinations of risk prediction approaches (AI, PRS or family history) and screening frequency for low-risk women (no screening or biennial screening). The other two strategies involved annual screening for all women and no screening, respectively. Data used to populate the model were sourced from the published literature.

**Results:**

Risk prediction using AI followed by no screening for low-risk women is the most cost-effective strategy. It dominates (i.e., costs more and generates fewer quality adjusted life years (QALYs)) strategies for risk prediction using PRS followed by no screening or biennial screening for low-risk women, risk prediction using AI or family history followed by biennial screening for low-risk women, and annual screening for all women. It also extendedly dominates (i.e., achieves higher QALYs at a lower incremental cost per QALY) the strategy for risk prediction using family history followed by no screening for low-risk women. Meanwhile, it is cost-effective versus no screening, with an incremental cost-effectiveness ratio of $23,755 per QALY gained.

**Conclusions:**

Risk prediction using AI followed by no breast cancer screening for low-risk women is the most cost-effective strategy. This finding can be explained by AI’s ability to identify high-risk women more accurately than PRS and family history (which reduces the possibility of delayed breast cancer diagnosis) and fewer false-positive diagnoses from not screening low-risk women.

**Supplementary Information:**

The online version contains supplementary material available at 10.1186/s12885-022-09613-1.

## Background

There is widespread debate among clinicians and researchers globally over what constitutes appropriate breast cancer screening, especially for women younger than age 50 [1]. Consequently, existing guidelines on mammography screening for breast cancer vary widely, even within a country. In the United States (US), the American College of Obstetricians and Gynecologists (ACOG) and the American College of Radiology (ACR) recommend annual mammography starting at age 40 for all women [2]. Meanwhile, the most recent US Preventive Services Task Force (USPSTF) guidelines recommend biennial mammography between ages 50 to 74 years for women without family history of breast cancer while indicating that women with family history may benefit from starting screening between ages 40 and 49 [2]. In Canada, breast cancer experts have challenged the Canadian Preventive Task Force which recommends against breast cancer screening for women aged between 40 and 49 years who are not at high risk, arguing that these recommendations are “outdated and dangerous” and have called for annual screening of all women above age 40 [3].

Cost-effectiveness analyses can inform this debate by estimating and comparing the costs and effectiveness of alternative screening strategies to identify the most cost-effective screening strategy. However, despite several cost-effectiveness analyses of alternative screening intervals and starting ages for mammography screening associated with current screening guidelines [4, 5], the results remain inconclusive. Earlier studies have found starting screening at age 40 was not cost-effective relative to starting at age 50 [4], which lends support to the existing USPSTF guidelines while more recent cost-effectiveness analyses point to the value of extending screening to women younger than age 50 [5] as recommended by ACOG/ACR.

A key limitation of existing guidelines is that these do not fully account for heterogeneity in women’s risk of breast cancer. For instance, while risk assessment tools may consider family history or breast density as risk factors, these tools do not consider the full set of genetic markers now known to be associated with breast cancer. Furthermore, breast density measurements are also subject to radiologists’ assessment and discernment. From an economic perspective, a more rigorous risk stratification can enable focusing health care resources on screening women with high risk while avoiding unnecessary screening and follow-up costs for those with low risk.

Two new risk prediction approaches have recently emerged, namely polygenic risk score (PRS) and artificial intelligence (AI). PRSs estimate a woman’s risk of breast cancer based on susceptibility loci identified through genome wide association studies [6]. AI algorithms, in contrast, identify discriminative image patterns from full-field mammograms to categorize a woman’s risk of developing breast cancer in the future [7].

To date, there is very little evidence on the cost-effectiveness of using these new risk-stratification approaches to aid breast cancer screening. Only one study has examined the cost-effectiveness of PRS-based risk-stratified mammography screening versus screening all women aged between 50 and 69 years and no screening for breast cancer. This study found that offering mammography screening only to women above the 70th percentile of the PRS-based risk distribution is cost-effective relative to screening all women aged between 50 and 69 years and no screening [8]. Notably, no study has compared the cost-effectiveness of risk-stratified mammography screening based on risk prediction using AI vs PRS. Our study fills this evidence gap.

In this study, we examine the cost-effectiveness of using AI or PRS to guide mammography screening for breast cancer compared with screening based exclusively on family history (similar to USPSTF guidelines), annual screening for all women (similar to ACOG/ACR guidelines) and no screening, among white women. As most of the debate over breast cancer screening centers on screening for women aged between 40 and 49 years and as data on predictive ability of AI has been validated only for the short-term [7], we consider AI and PRS for guiding screening for only women in the 40 to 49 years age group, with screening for older women based on existing guidelines.

## Methods

### Study cohort and risk of breast cancer

Our model simulated 100,000 white women aged 40 years with no previous history of breast cancer. Each woman had an underlying risk of developing breast cancer based on a recent risk distribution estimated for US white females using a comprehensive set of genetic and other non-modifiable and modifiable breast cancer risk factors [9]. As criteria for who is considered ‘high risk’ for screening purposes differ across guidelines, we conservatively classified women into three categories: (i) ‘true’ low risk, defined as those with an underlying risk of breast cancer less than 1.1 times the average risk in the population of 40 year old women (that is, relative risk (RR) is lower than 1.1); (ii) ‘true’ high risk, defined as those with RR between 1.1 and 4; and (iii) ‘true’ very high risk, defined as those with RR of 4 or higher. The RR threshold of 1.1 was chosen because it can capture a broad range of factors known for increasing risk of breast cancer, including family history of breast cancer, reproductive risk factors, genetic variations and dense breast on mammography [10]. Meanwhile, the RR threshold of 4 captures factors such as history of chest radiation and atypical hyperplasia [11, 12]. With these RR thresholds, 1% of our hypothetical study cohort was classified as ‘true’ very high risk, 42% as ‘true’ high risk and the remaining 57% as ‘true’ low risk.

### Screening strategies

**Fig. 1:**
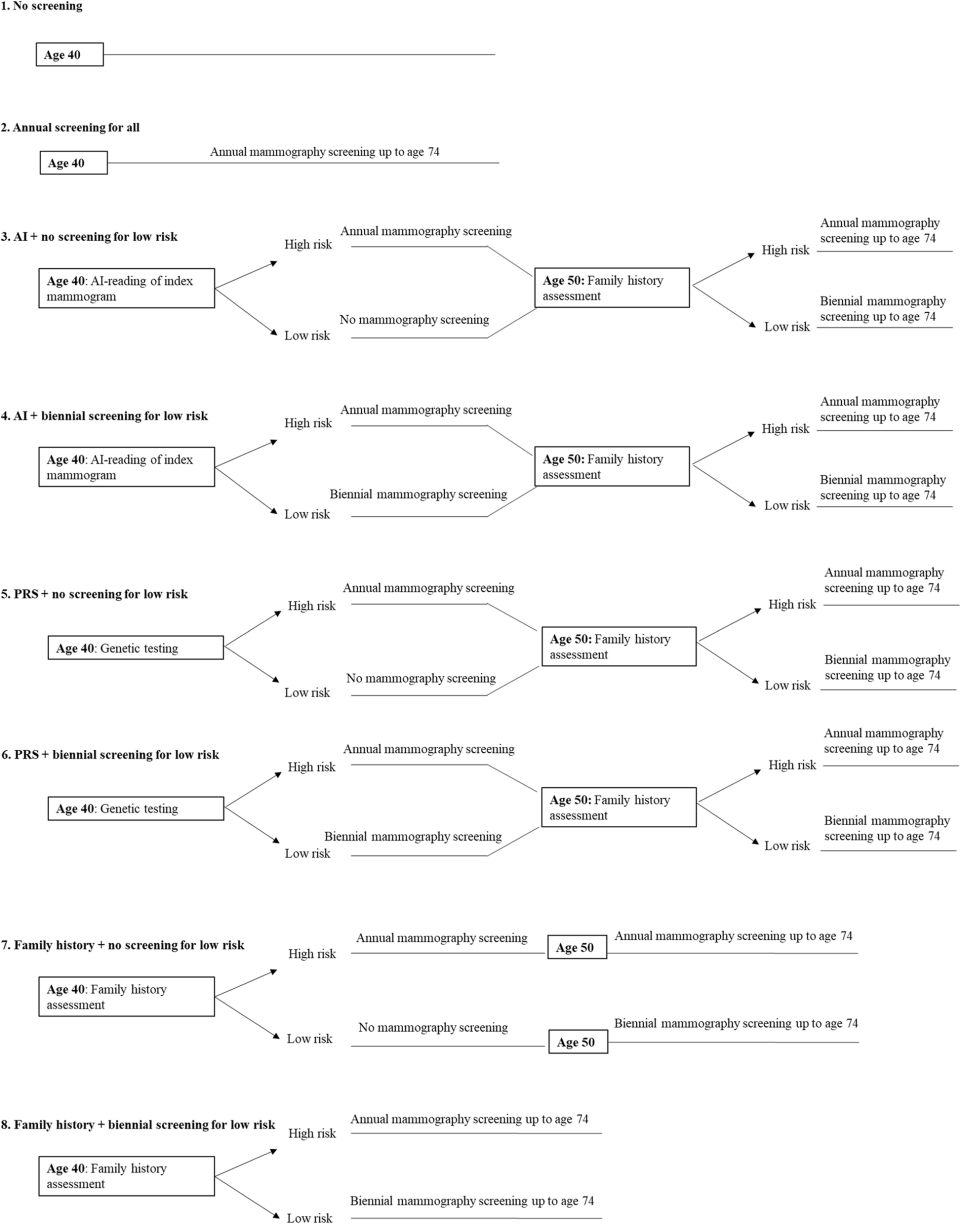
Screening strategies. In strategies 3–6, ‘High risk’ and ‘Low risk’ during age 40–49 refer to estimated high-risk and low-risk by AI or PRS, while beyond age 50 refer to presence or absence of family history, respectively. In strategies 7–8, ‘High risk’ and ‘Low risk’ refer to presence or absence of family history, respectively. Beyond age 50, in all strategies except strategies 1 and 2, women without family history undergo biennial screening; those with family history undergo annual screening. Screening in all strategies ceases at age 74

We compared eight alternative screening strategies as shown in Fig. 1. The first two strategies involved no screening and annual screening for all women, respectively. The remaining six strategies were defined by combinations of risk prediction approaches (AI, PRS or family history) and screening frequencies among low-risk women aged 40–49 (no screening or biennial screening). We describe these strategies in detail below.

#### No screening

In strategy 1 (‘No screening’, hereafter), women were not screened at any age regardless of risk level.

#### Annual screening for all

In strategy 2 (‘Annual screening for all’, hereafter), all women (regardless of risk level) underwent annual mammography starting at age 40, similar to recommendations by ACOG and ACR.

#### Screening guided by AI

Strategies 3 and 4 involved risk stratification based on AI reading of an index mammogram. All women underwent an index mammogram at age 40, which was interpreted using AI to predict risk of breast cancer. This mammogram may or may not be part of standard screening services. Women predicted to have high risk (RR > =1.1) underwent annual digital mammography starting at age 40. In strategy 3 (‘AI + no screening for low-risk’, hereafter), women predicted to have low risk were not screened while in strategy 4 (‘AI + biennial screening for low-risk’, hereafter), they underwent biennial screening. This screening pattern continued until age 49. Beyond age 50, screening followed the existing USPSTF guideline as described below.

#### Screening guided by PRS

In strategies 5 (‘PRS + no screening for low-risk’, hereafter) and 6 (‘PRS + biennial screening for low-risk’, hereafter), screening pathways were the same as in strategies 3 and 4; however, risk stratification was performed using PRS instead of AI. All women underwent genetic testing at age 40 in which 76 single nucleotide polymorphisms (SNPs) known to be associated with breast cancer were genotyped [6].

#### Screening guided by family history

In strategies 7 and 8, screening was guided by family history (similar to existing recommendations by the USPSTF). For women aged between 40 and 49 years, existing USPSTF recommendation to screen women without family history is only a grade C recommendation (i.e., the net benefit of screening in this group is small) [11, 13]. Therefore, in strategy 7 (‘Family history + no screening for low-risk’, hereafter), we considered that women younger than age 50 without family history were not screened, while in strategy 8 (‘Family history + biennial screening for low-risk’, hereafter), they were screened biennially. The USPSTF guidelines indicate that women with family history may benefit from starting screening before age 50 [2] but do not specify frequency of screening for these women. Given that most other screening guidelines recommend annual screening for high-risk women [2], we considered that women with family history underwent annual mammography starting at age 40.

Beyond age 50, screening in strategies 3-8 followed existing USPSTF guidelines. Therefore, women without family history were screened biennially [11]. Furthermore, as the USPSTF does not specify screening frequency for those with family history, similar to younger women, women with family history underwent annual mammography. In all strategies (except ‘No screening’), screening ceased at age 74.

The eight strategies, thus, differed in the proportion of women subjected to aggressive screening. ‘Annual screening for all’ was the most aggressive as all women, including those at low risk, were screened annually starting at age 40. By contrast, in the remaining strategies, low-risk women younger than age 50 were either not screened or screened only biennially while those aged over 50 were screened biennially. While screening frequencies were the same in strategies 3,5 and 7, and in strategies 4, 6 and 8, these strategies differed in their accuracy of risk prediction for women aged between 40 and 49, which in turn determined the proportion of women screened prior to age 50.

### Model structure

We developed a hybrid decision tree/microsimulation model to estimate the costs and effectiveness of the eight screening strategies. The analysis was conducted from the health care system’s perspective. Cycle length was 1 year and lifetime horizon was used.

**Fig. 2:**
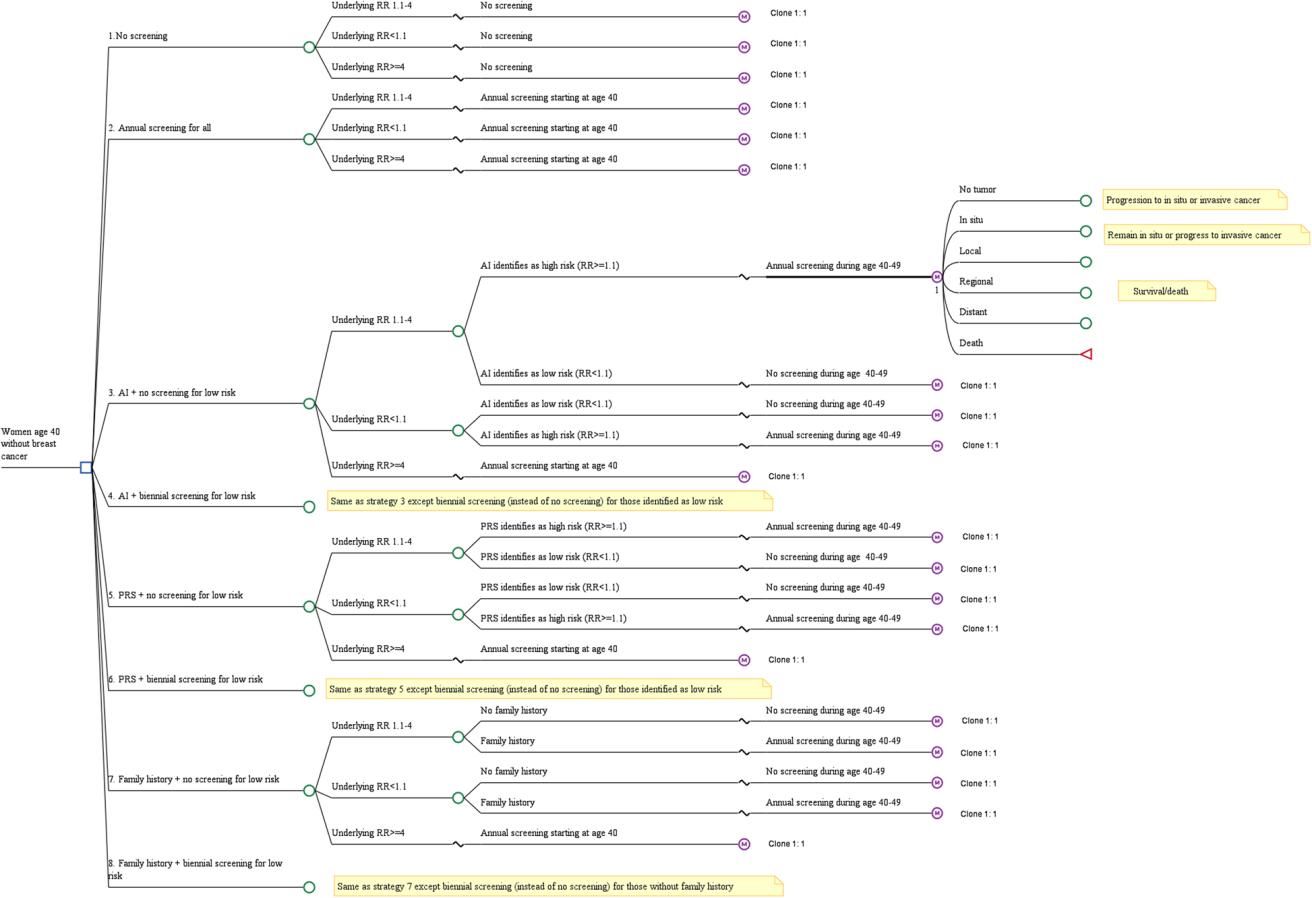
Simplified depiction of model. Clinical pathways for strategies 4, 6 and 8 are the same as for strategies 3, 5 and 7, respectively, except that patients identified as low risk are screened biennially instead of no screening. Clinical pathways for progression to in situ or invasive cancer and to death follow the pathways described in Schousboe et al, 2011 [14]). Beyond age 50, in all strategies except strategies 1 and 2, women without family history undergo biennial screening; those with family history undergo annual screening

Figure 2 shows a simplified depiction of the model. The decision tree component of the model captured risk prediction and stratification at age 40 based on AI, PRS or family history. Women entering the model had an underlying ‘true’ low, high or very high risk of breast cancer. As risk factors associated with very high risk (RR > =4) are likely known a priori, women with RR > =4 did not require risk prediction and underwent annual screening regardless of screening strategy (except in the ‘No screening’ strategy). Depending on risk-stratification strategy, AI, PRS or family history were used to predict the underlying risk for the remaining women; the extent to which the estimated risk category matched the underlying risk category was determined by the accuracy of each method (described below).

The microsimulation component, which was adapted from a previously published model [14], simulated the screening, diagnosis, disease progression and mortality from breast cancer. All women entering the microsimulation model had no tumor but could develop in-situ or invasive cancer over time based on observed age-specific incidence rates; in situ cancer could further progress to invasive cancer. Invasive cancers were classified into local, regional and distant stages [14]. Women who underwent mammography screening were more likely to be diagnosed with in situ cancer. However, more aggressive mammography screening also resulted in more cancers being diagnosed in earlier (instead of more advanced) stages [14]. Women who developed invasive breast cancer faced risk of death from cancer or from other causes.

### Model inputs

Inputs used in our model are presented in Table 1 and described below. Further details are provided in the Online Supplementary Materials.

**Table 1:** Model Inputs

Variable	Value	Source
**Risk prediction**		
AI (AUC)	0.71	[7]
PRS (AUC)	0.69	[6]
Family history (proportion correctly identified as high risk)	0.37	Authors’ calculation based on [15, 16]
**Stage distributions**		
***No screening***		
Local	0.517	[17]
Regional	0.436 (age < 50); 0.401 (age > =50)	
Distant	0.047 (age < 50); 0.082 (age > =50)	
***Biennial screening***		
Local	0.650 (age 40–49); 0.690 (age 50–59); 0.742 (age 60–69); 0.758 (age 70–74)	[14, 18]
Regional	0.341 (age 40–49); 0.303 (age 50–59); 0.252 (age 60–69); 0.237 (age 70–74)	
Distant	0.009 (age 40–49); 0.007 (age 50–59); 0.006 (age 60–69); 0.005 (age 70–74)	
***Annual screening***		
Local	0.683 (age 40–49); 0.696 (age 50–59); 0.732 (age 60–69); 0.772 (age 70–74)	[14, 18]
Regional	0.310 (age 40–49); 0.297 (age 50–59); 0.262 (age 60–69); 0.223 (age 70–74)	
Distant	0.007 (age 40–49); 0.007 (age 50–59); 0.006 (age 60–69); 0.005 (age 70–74)	
**Probabilities**		
ER positive, HER2 negative	0.76	[10]
ER positive, HER2 positive	0.1	
ER negative, HER2 positive	0.04	
ER negative, HER2 negative	0.1	
**Mortality hazard reduction**		
Tamoxifen	0.67	[19]
Trastuzumab	0.66	[19]
***Sensitivity and specificity of mammography***		
Sensitivity	0.824 (age 40–49); 0.805 (age 50–59); 0.899 (age 60–69); 0.86 (age 70–74)	[20]
Specificity	0.88 (age 40–49); 0.909 (age 50–59); 0.921 (age 60–69); 0.928 (age 70–74)	
**Costs ($)**		
AI	112 (28)	[21], Author’s calculation
OncoArray genetic test	115 (29)	[22]
Genetic counseling (per session)	44 (11)	[23]
Mammography	152 (38)	[24]
Additional diagnostic costs (true positive diagnosis)		
Age 40–49	2491 (623)	[25]
Age 50–64	2337 (584)	
Age 65–74	2350 (588)	
Additional diagnostic costs (false positive diagnosis)		
Age 40–49	261 (65)	[25]
Age 50–64	309 (77)	
Age 65–74	310 (77)	
Treatment costs		
In situ, initial cost	11,543 (2886); 10,329 (2582)	[14, 19]
In situ, continuing cost	0	
Localized, initial cost	29,374 (7343); 18,995 (4749)	
Localized, continuing cost	1986 (497); 1267 (317); 1210 (303); 1446 (362); 1044 (261); 817 (204)	
Localized, terminal cost	51,800 (12950)	
Regional, initial cost	51,859 (12965); 35,365 (8841)	
Regional, continuing cost	6747 (1687); 4572 (1143); 4315 (1079); 3744 (936); 2662 (666); 2353 (588)	
Regional, terminal cost	58,172 (14543)	
Distant, initial cost	56,702 (14176); 43,543 (10886)	
Distant, continuing cost	23,581 (5895); 20,945 (5236); 20,162 (5040); 17,744 (4436); 13,094 (3274); 13,478 (3370)	
Distant, terminal cost	73,970 (18493)	
Tamoxifen (5 years)	1519 (76)	[19]
Trastuzumab	81,717 (20429)	[19]
**Utilities**		
Disutility from screening	0.006 (0.00003) for 1 week	[25]
Disutility from additional diagnosis	0.105 (0.00001) for 5 weeks	
Health state		
Healthy	0.762–0.859 (depending on age and time since diagnosis)	[14]
In situ	0.689–0.777 (depending on age and time since diagnosis)	
Local	0.645–0.842 (depending on age and time since diagnosis)	
Regional	0.574–0.777 (depending on age and time since diagnosis)	
Distant	0.574–0.715 (depending on age and time since diagnosis)	

All costs are in 2020 US dollars ($). Standard deviations used in probabilistic sensitivity analyses are in parentheses. Calculations by European Society of Radiology suggest fixed costs of €60,000 ($65,300 @ €1 = US$1.08 [26]) for AI technology in addition to €20,000 ($21,770) annually for the software license [21]. Assuming equipment is amortized in 10 years, and with 8695 mammogram facilities in the US [27] serving over 2 million women aged 40 years [28], cost of AI reading of each mammogram amounts to ~$112. Initial treatment costs for each stage are for age < 70 and age > =70, respectively, calculated as the weighted average of costs of different breast cancer treatments with proportion of patients receiving each type of treatment as the weight [19]. Continuing treatment costs for each stage are for 1 to 5 and > =6 years after the year of diagnosis, respectively. AJCC stage-specific costs reported in [19] were converted to SEER stage-specific costs using proportions reported in Schousboe et al. [14]

#### Accuracy of risk prediction

The key determinant of costs and effectiveness of each screening strategy was the accuracy of risk prediction. Higher accuracy of risk prediction implied that fewer women with underlying high-risk were incorrectly predicted to be at low risk, resulting in timely diagnosis and treatment of cancer for high-risk women. It also meant that fewer low-risk women were incorrectly predicted to be at high risk, leading to reduction in screening and fewer false-positive diagnoses.

In our model, accuracy of breast cancer risk prediction using AI and PRS was measured using area under the receiver operating characteristic curve (AUC) obtained from published studies [6, 7]. As real-world clinical decisions will also likely utilize information on other demographic and personal risk factors (such as weight, family history and breast density) in addition to AI or PRS, we used AUC values for models based on both AI or PRS and other risk factors. Using data from digital screening mammograms read by deep learning algorithms (AI), information on other demographic and personal risk factors and breast cancer outcomes from tumor registries, Yala et al. estimated an AUC of 0.71 for white females in the US [7]. We chose this study to obtain the AUC for AI owing to its large study sample of patients seen in the US (over 31,000 patients in the training dataset and over 3900 patients in the test set) [7]. Meanwhile, AUC for PRS was obtained from Vachon et al., a recent, high-quality study that estimated the AUC for PRS combined with other risk factors for a large study sample primarily consisting of American women [6]. Vachon et al. estimated an AUC of 0.69 for a model that combined PRSs developed based on 76 SNPs and information from the Breast Cancer Surveillance Consortium (BCSC) five-year risk-prediction model [6]. We followed a previously published method to simulate distributions of RR estimated using AI or PRS using these AUC values [29, 30]. Women with estimated RR of 1.1 or higher were then classified as high risk while those with estimated RR below 1.1 as low risk. We note that as AUC of both AI and PRS is below 1, not all ‘true’ high risk women will be correctly classified as such.

In strategies that involved risk prediction based on family history, as women with an underlying low risk will not have a family history of breast cancer, all low-risk women will be correctly classified as such. Among high-risk women, we assumed that 37% will be correctly classified. This proportion was calculated as the share of US women with first-degree family history of breast cancer (16% [15, 16]) among high-risk women (43% of our study cohort).

#### Incidence and stage distribution of breast cancer and mortality risk

To estimate a woman’s likelihood of developing in situ or invasive breast cancer, we multiply age-specific breast cancer incidence rates per 100,000 white women in the US [31] (adjusted for increase in incidence rates due to mammography screening [14]) with the woman’s ‘true’ RR. The stage at cancer detection depended on screening frequency and sensitivity of mammography; the latter depended on patient age and was obtained from the published literature [20]. Women receiving more aggressive screening were diagnosed at earlier stages than those receiving less frequent screening. Stage distribution at diagnosis in the absence of screening was calculated based on the proportions of local, regional and distant cancers observed among white women aged below or above 50 years during 1975–1979 (when mammography screening was not widespread in the US) [17]. Meanwhile, stage distributions with annual or biennial screening were obtained from more recent estimates based on 1996–2012 Breast Cancer Surveillance Consortium data [18]. Patients diagnosed with invasive breast cancer faced risk of breast cancer mortality for up to 20 years after diagnosis. This risk was specific to age and stage at diagnosis as well as estrogen-receptor (ER) and human epidermal growth factor 2 (HER2) status [32]. All women faced risk of mortality from non-breast cancer causes which was age-specific, and was obtained by subtracting age-specific breast cancer mortality from the 2017 US life tables [33].

#### Costs

The cost of each strategy included cost of risk prediction (index mammogram read by AI technology or genetic testing as applicable), cost of screening with digital mammogram (if any), and cost of breast cancer treatment determined by the stage at cancer diagnosis (treatment costs were lower for cancers detected at an earlier stage). Cost of genetic test to determine PRSs was the cost of OncoArray test in US laboratories [22]. We assumed that patients underwent genetic counselling before and after the genetic test, and that each counselling session costed $44 [23]. While cost of AI-based risk prediction in clinical practice is not yet available, calculations by European Society of Radiology suggest fixed costs of €60,000 ($65,300) in addition to an annual cost of €20,000 ($21,770) for the software license [21]. Assuming equipment is amortized in 10 years, and with 8695 mammogram facilities in the US [27] serving nearly two million women aged 40 years [28], cost of AI reading of each mammogram amounts to ~$112. We varied cost of AI reading per mammogram over a wide range (up to $500) in the sensitivity analyses.

The cost of mammogram was obtained from Center for Medicare and Medicaid’s 2020 Physician Fee Schedule [24]. Cost of diagnostic work-up following a positive diagnosis and cost of treatment of breast cancer were obtained from the published literature [19, 25]. All costs were estimated in 2020 US dollars and discounted at 3% per year [34].

#### Effectiveness

Effectiveness was measured in terms of Quality Adjusted Life Years (QALYs) that captured a person’s life expectancy adjusted by his/her health-related quality of life called utility. Screening entailed disutility of 0.006 QALYs for 1 week and diagnostic workup following a positive screening result involved disutility of 0.105 QALYs for 5 weeks [25]. Utilities were specific to patient age and stage of cancer [14]. For all cancer stages, utilities in the first year after breast cancer diagnosis were lower than in later years [14]. All utility values were discounted at 3% per year [34].

### Cost effectiveness analysis

We estimated the total costs and QALYs of the eight strategies. A strategy was considered cost-effective relative to another strategy if the Incremental Cost Effectiveness Ratio (ICER), calculated as the difference between the overall costs of the two strategies divided by the difference between the total QALYs gained, was lower than the conventional willingness-to-pay threshold (WTP) of $100,000 per QALY. Meanwhile, a strategy was dominated if it was both more costly and less effective than the other strategy or extended dominated if it achieved fewer total QALYs than a more costly strategy at a higher incremental cost per QALY (i.e., its ICER relative to the next less costly strategy was higher than the ICER of a more effective strategy) [35].

In addition to the eight strategies examined in the main analysis, we conducted an augmented analysis which included 4 additional strategies. These additional strategies were similar to strategies 3–6 above, except that risk prediction was performed exclusively using AI or PRS, i.e., without considering demographic and personal risk factors. Thus, AUC values in these additional strategies were 0.69 for AI [7] and 0.63 for PRS [36] (instead of 0.71 and 0.69, respectively, in strategies 3-6).

Furthermore, we conducted several sensitivity analyses. First, we varied values of key costs and utilities in one-way sensitivity analyses and addressed parameter uncertainty using probabilistic sensitivity analyses (PSA). Next, we varied AUCs of AI and PRS to 20% lower and higher values than those used in the main analysis.

We also examined the robustness of our findings to the choice of the RR threshold used to define estimated high risk. Following previous studies, we used alternative thresholds of 1.3 and 2 (instead of 1.1 used in the base case analysis) [37]. All analyses were performed using TreeAge Pro 2019 v2.1 [38].

### Model validation

We assessed the validity of our model following the Assessment of the Validation Status of Health-Economic decision models (AdViSHE) tool [39] and guidelines of the International Society for Pharmacoeconomics and Outcomes Research [40]. First, we conducted trace analysis and compared the modelled lifetime cumulative breast cancer incidence and mortality with screening to recently observed proportions. Next, while cost-effectiveness of AI-based screening has not been examined previously, we cross-validated the estimated incremental costs, QALYs and false-positive rates (compared with no screening) against previous studies for the strategy where risk prediction is based on family history and those without family history are screened biennially starting at age 50.

## Results

### Base case analysis

Table 2 summarizes the lifetime costs and QALYs gained, and breast cancer outcomes with each screening strategy. ‘No screening’ involved the least lifetime total costs ($1.75 billion per 100,000 women) but also generated the fewest QALYs (1,976,720 per 100,000 women). The strategies involving screening resulted in $77.8 million - $276.3 million higher lifetime costs (per 100,000 women) and 1521–4110 additional QALY (per 100,000 women) relative to ‘No screening’.

**Table 2:** Lifetime Costs, QALYs and Breast Cancer Outcomes by Screening Strategy

Strategy	Cost (in 1000 $)	Effectiveness (in QALYs)	No. (%) of true high risk women classified as high risk	No. (%) of true low risk women classified as low risk	No. of breast cancer deaths (per 100,000 women)	No. of false positive diagnoses (per 100,000 women)
No screening	1,745,808	1,976,720	–	–	3367	0
Family history + no screening for low risk	1,823,664	1,978,241	15,461 (36.0)	56,444 (100)	2988	121,737
AI + no screening for low risk	1,843,441	1,980,830	24,525 (57.0)	49,122 (87.0)	2956	141,339
PRS + no screening for low risk	1,852,227	1,980,713	24,381 (56.7)	48,805 (86.4)	2936	141,443
Family history + biennial screening for low risk	1,879,254	1,980,731	15,461 (36.0)	56,444 (100)	2916	170,917
PRS + biennial screening for low risk	1,909,968	1,978,418	24,381 (56.7)	48,805 (86.4)	2885	180,219
AI + biennial screening for low risk	1,910,153	1,978,604	24,525 (57.0)	49,122 (87.0)	2903	180,163
Annual screening for all	2,022,120	1,978,717	–	–	2778	290,325

All costs are in 2020 US dollars ($). Costs and effectiveness are calculated per 100,000 women. All strategies (except ‘No screening’) involve annual screening for women identified as high-risk. ‘AI’ refers to risk prediction accounting for both AI and other risk factors. ‘PRS’ refers to risk prediction accounting for both PRS and other risk factors. Beyond age 50, women without family history are screened biennially and those with family history are screened annually in all strategies except ‘No screening’ and ‘Annual screening for all’ strategies. ‘No. of false positive diagnoses’ refers to total number of false positive diagnoses among all mammograms performed during the lifetimes of 100,000 women. As specificity of each mammogram is < 100%, a woman can have more than one false-positive diagnosis in her lifetime

**Fig. 3:**
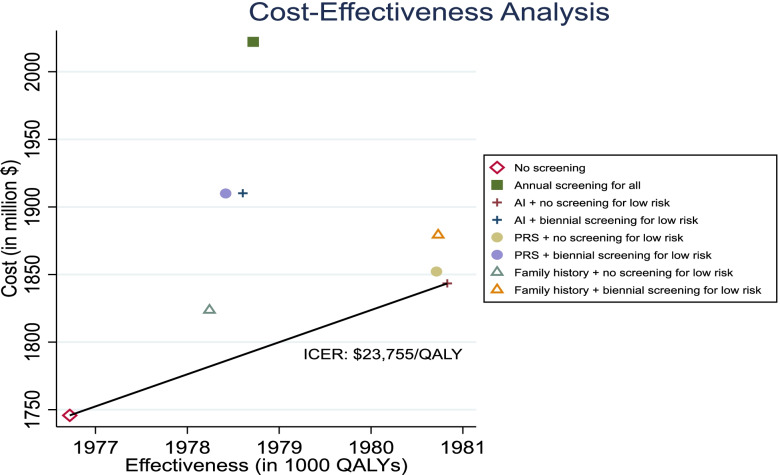
Cost-effectiveness plane. ICER: Incremental Cost-Effectiveness Ratio

The cost-effectiveness plane in Fig. 3 shows the results from stepwise comparisons with the next less costly strategy. Among the eight strategies, only ‘No screening’ and ‘AI + no screening for low-risk’ strategies lay on the cost-effectiveness efficiency frontier. The ‘Family history + no screening for low-risk’ strategy was extended dominated while the remaining 5 strategies (that involved either risk prediction using PRS and/or biennial or annual screening for low-risk women) were dominated by ‘AI + no screening for low-risk’. Excluding these dominated and extended dominated strategies, ‘AI + no screening for low-risk’ was the most cost-effective strategy. It cost $97.6 million (per 100,000 women) more than ‘No screening’ but generated 4110 additional QALYs (per 100,000 women). The ICER compared with ‘No screening’ was $23,755 per QALY gained which was lower than the conventional WTP threshold of $100,000 per QALY gained.

The superior cost-effectiveness of ‘AI + no screening for low-risk’ compared with other screening strategies is explained by the combination of: (i) higher accuracy of AI in identifying high-risk women compared with family history and PRS; and (ii) prevention of costs and disutility of screening and additional diagnostic work-up for low-risk women. As shown in Table 2, AI correctly classifies 57% of true high-risk women as such, compared with 36% with family history. Consequently, even though total costs of ‘AI + no screening for low risk’ are higher than ‘Family history + no screening for low risk’ (because more women are screened during ages 40 to 49), more high-risk women would benefit from this screening, as reflected in fewer breast cancer deaths (2956 vs 2988 per 100,000 women). While risk prediction using AI is also more costly than genetic testing, its higher accuracy justifies the higher cost: 57% vs 56.7% high-risk women and 87% vs 86.4% of low-risk women are correctly classified with AI and PRS, respectively. The lower accuracy of PRS implies that more low-risk women incorrectly undergo annual screening between ages 40 and 49 compared with AI, leading to more false-positive diagnoses (141,443 per 100,000 women with ‘PRS + no screening for low risk’ vs 141,339 per 100,000 women with ‘AI + no screening for low risk’).

Meanwhile, no screening for low-risk women aged 40–49 explain the lower costs and higher effectiveness of this strategy relative to strategies involving biennial or annual screening for low-risk women. Even though breast cancer deaths are higher as not all women are screened during age 40–49, there are 17–51% fewer false-positive diagnoses. Thus, not screening women identified as low-risk saves both costs and disutility of screening and additional diagnostic work-up.

The results from the augmented analysis (that included the 4 additional strategies for risk prediction using AI or PRS without other risk factors) supported our base case findings. As shown in Table A1 (Online Supplementary Materials), ‘AI + no screening for low risk’ remained the most cost-effective strategy. In particular, it dominated the strategies involving risk prediction based exclusively on AI or PRS.

### Sensitivity analyses

**Fig. 4:**
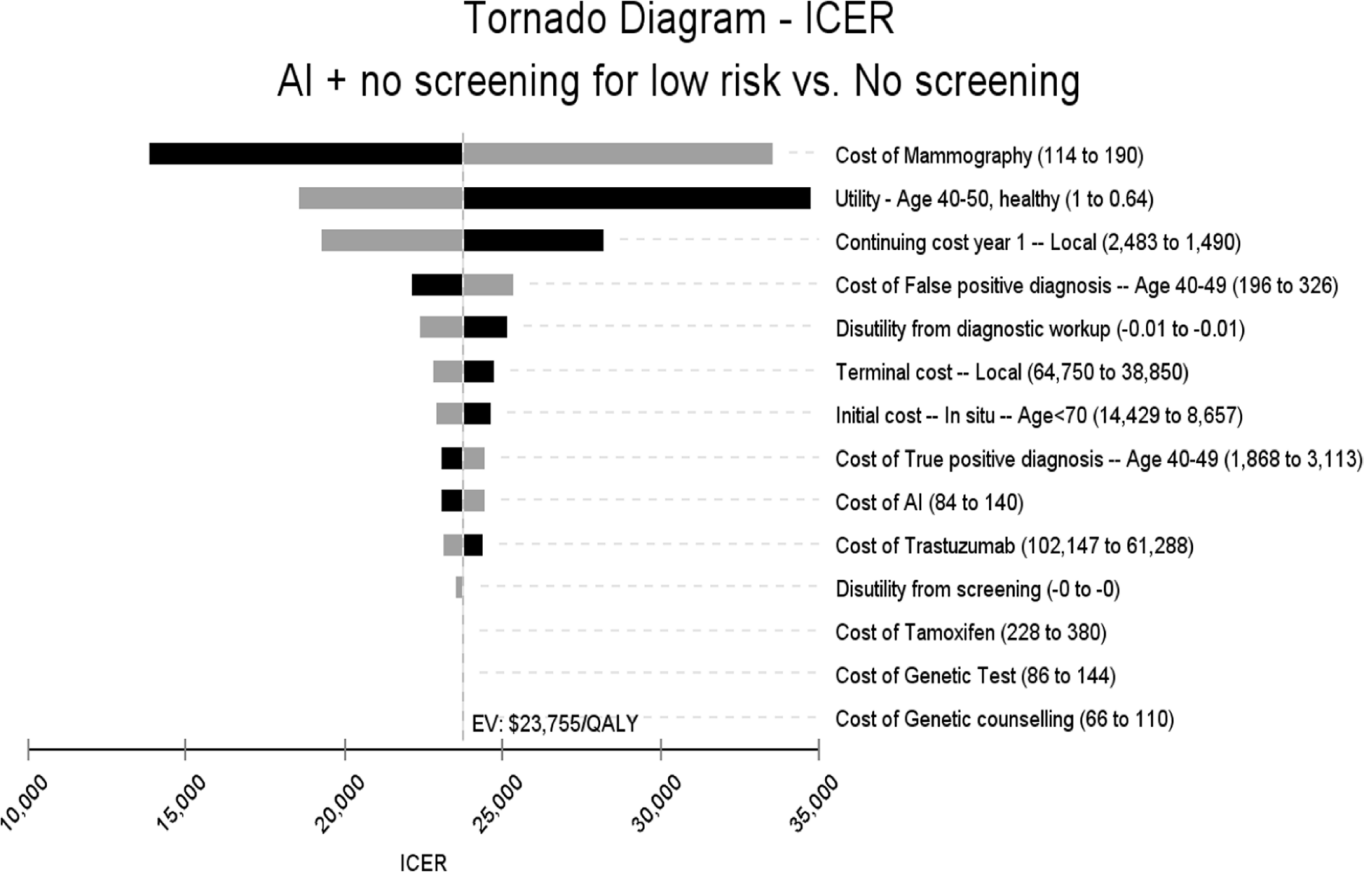
Tornado Diagram. Costs and utilities are varied in a range of ±25% of base case values. ICER: Incremental Cost-Effectiveness Ratio

**Fig. 5:**
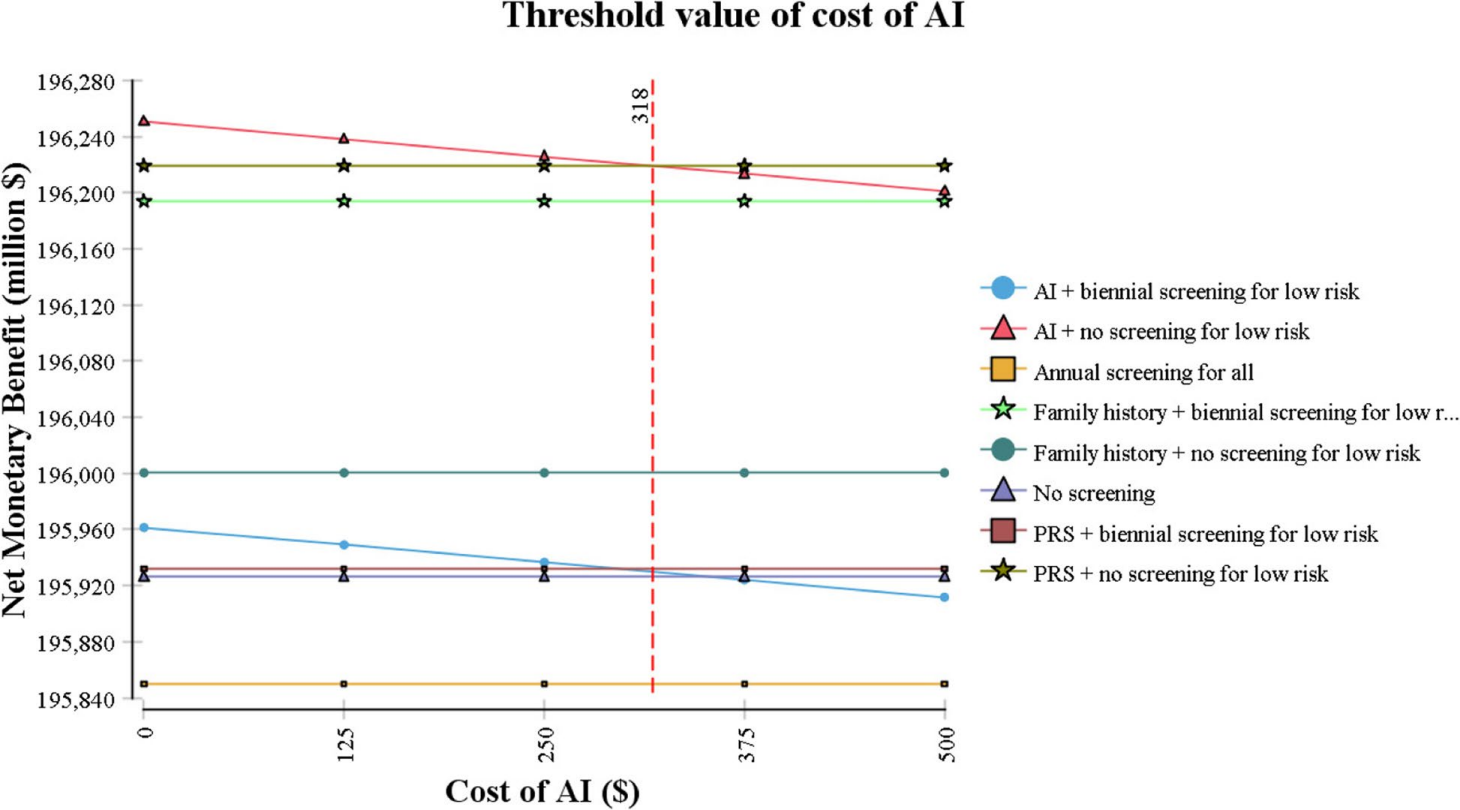
Threshold analysis for cost of AI

**Fig. 6:**
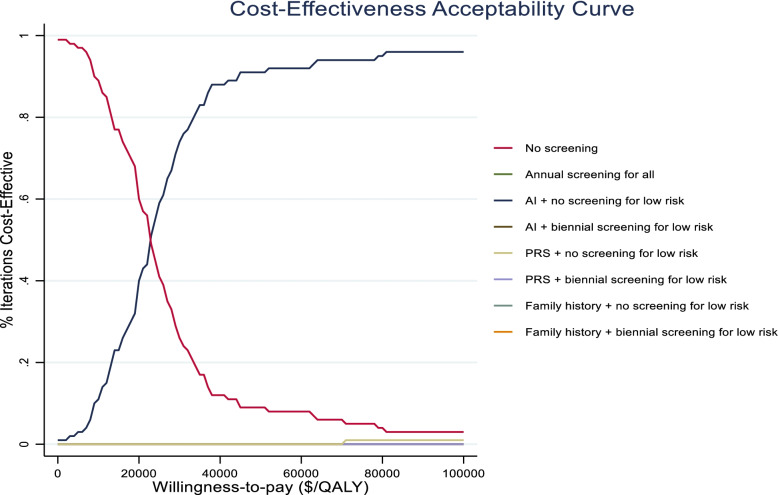
Cost-effectiveness acceptability curve

The results from one-way sensitivity analyses are presented in a tornado diagram in Fig. 4. They indicate that the ICER is most sensitive to cost of mammography and health state-specific utilities and costs. For all values of these costs and utilities in the ±25% range, however, ‘AI + no screening for low-risk’ remains cost-effective vs no screening. In particular, it remains the most cost-effective screening strategy as long as cost of AI reading is below $318 per mammogram (Fig. 5). The cost-effectiveness acceptability curve shows that, at the WTP threshold of $100,000/QALY, ‘AI + no screening for low risk’ is cost-effective in 96% of iterations (Fig. 6).

Table 3 presents results from additional sensitivity analyses. It shows that ‘AI + no screening for low risk’ remained the most cost-effective strategy even when we used AUC values for AI and PRS that were 20% lower or higher than that in the base case.

**Table 3:** Sensitivity analyses

Strategy	Cost (in 1000 $)	Effectiveness (in QALYs)	ICER ($/QALY)
***Panel A: AUC 20% lower than base case***			
No screening	1,745,808	1,976,720	–
Family history + no screening for low risk	1,823,664	1,978,241	Ext. dominated
PRS + no screening for low risk	1,839,375	1,980,821	22,819
AI + no screening for low risk	1,841,639	1,980,909	25,752
Family history + biennial screening for low risk	1,879,254	1,980,731	Dominated
PRS + biennial screening for low risk	1,914,138	1,978,829	Dominated
AI + biennial screening for low risk	1,920,087	1,978,752	Dominated
Annual screening for all	2,022,120	1,978,717	Dominated
***Panel B: AUC 20% higher than base case***			
No screening	1,745,808	1,976,720	–
Family history + no screening for low risk	1,823,664	1,978,241	Ext. dominated
AI + no screening for low risk	1,841,419	1,981,155	21,558
PRS + no screening for low risk	1,851,053	1,980,534	Dominated
Family history + biennial screening for low risk	1,879,254	1,980,731	Dominated
PRS + biennial screening for low risk	1,916,570	1,978,280	Dominated
AI + biennial screening for low risk	1,918,578	1,978,684	Dominated
Annual screening for all	2,022,120	1,978,717	Dominated

ICER: Incremental Cost-Effectiveness Ratio. All costs are in 2020 US dollars ($). Costs and effectiveness are calculated per 100,000 women. All strategies (except ‘No screening’) involve annual screening for women identified as high-risk. ‘AI’ refers to risk prediction accounting for both AI and other risk factors. ‘PRS’ refers to risk prediction accounting for both PRS and other risk factors. Beyond age 50, women without family history are screened biennially and those with family history are screened annually in all strategies except ‘No screening’ and ‘Annual screening for all’ strategies. In Panel A, ICER for ‘AI + no screening for low risk’ is calculated relative to ‘PRS + no screening for low risk’. In Panel B, ‘Family history + no screening for low risk’ is extended dominated. Hence, ICER for ‘AI + no screening for low risk’ is calculated relative to ‘No screening’

### Alternative RR thresholds

In our base case analysis, we used an RR threshold of 1.1 to define estimated high risk with AI or PRS. However, this RR threshold is likely to itself be a policy alternative to be determined by decision-makers. We, therefore, conducted additional analyses in which we used alternative thresholds of 1.3 and 2 (instead of 1.1 used in the base case analysis) (Table 4) [37]. We found that, for higher RR thresholds, ‘PRS + no screening for low risk’ strategy generated higher QALYs but it also resulted in higher total costs than ‘AI + no screening for low-risk’, yielding an ICER that exceeded the WTP threshold of $100,000/QALY. Thus, ‘AI + no screening for low risk’ was still the optimal strategy.

**Table 4:** Alternative RR thresholds

Strategy	Cost (in 1000 $)	Effectiveness (in QALYs)	ICER ($/QALY)
***Panel A: RR threshold 1.3***			
No screening	1,745,808	1,976,720	–
Family history + no screening for low risk	1,823,664	1,978,241	Ext. dominated
AI + no screening for low risk	1,845,197	1,980,697	24,994
PRS + no screening for low risk	1,861,378	1,980,799	157,870
Family history + biennial screening for low risk	1,879,254	1,980,731	Dominated
PRS + biennial screening for low risk	1,921,493	1,978,380	Dominated
AI + biennial screening for low risk	1,936,713	1,978,375	Dominated
Annual screening for all	2,022,120	1,978,717	Dominated
***Panel B: RR threshold 2***			
No screening	1,745,808	1,976,720	–
AI + no screening for low risk	1,813,821	1,980,868	16,398
Family history + no screening for low risk	1,823,664	1,978,241	Dominated
PRS + no screening for low risk	1,830,198	1,980,922	299,464
Family history + biennial screening for low risk	1,879,254	1,980,731	Dominated
AI + biennial screening for low risk	1,906,031	1,979,063	Dominated
PRS + biennial screening for low risk	1,914,734	1,978,709	Dominated
Annual screening for all	2,022,120	1,978,717	Dominated

Note: ICER: Incremental Cost-Effectiveness Ratio. All costs are in 2020 US dollars ($). Costs and effectiveness are calculated per 100,000 women. All strategies (except ‘No screening’) involve annual screening for women identified as high-risk. ‘AI’ refers to risk prediction accounting for both AI and other risk factors. ‘PRS’ refers to risk prediction accounting for both PRS and other risk factors. Beyond age 50, women without family history are screened biennially and those with family history are screened annually in all strategies except ‘No screening’ and ‘Annual screening for all’ strategies. In Panel A, ‘Family history + no screening for low risk’ is extended dominated. Hence, ICER for ‘AI + no screening for low risk’ is calculated relative to ‘No screening’

### Model validation

Trace analysis indicated that the modelled lifetime cumulative breast cancer incidence and mortality were 16% and 2.9% with screening. These proportions were similar to the proportions observed for white women in 2016–18 reported by SEER (13% and 2.5%, respectively) [41]; the slight difference can be explained by < 100% adherence to screening guidelines in the real world [10]. Cross-validation against previous studies showed that our estimated incremental costs and QALYs for the ‘Family history + no screening for low risk’ strategy were similar to those estimated in a recent, high-quality cost-effectiveness analysis [19]: $778 vs $682 (in 2020 $) incremental costs and 0.015 vs 0.017 incremental QALYs. Furthermore, estimated number of false positive diagnoses for this strategy (121,737 per 100,000 women) fell within the range indicated by USPSTF (830–1325 per 1000 women) [11].

## Discussion

Our study provides the first cost-effectiveness analysis of using AI or PRS to risk-stratify 40–49 year old white women for breast cancer screening versus screening based on family history, annual screening for all women, and no screening. We found that risk prediction using AI followed by no screening for low-risk women is the most cost-effective strategy with an ICER of $23,755 per QALY gained.

Our results reveal several interesting patterns. We find that with both the AI and PRS algorithms, there exists a negative dose response relationship between screening frequencies and effectiveness, i.e., no screening is more effective than biennial screening. However, this pattern is reversed: (i) when risk prediction is based on family history; and (ii) when we compare ‘Annual screening for all’ with ‘AI/PRS + biennial screening for low risk’. These opposite patterns highlight how the accuracy of a risk prediction tool may reinforce or attenuate the effects of screening frequency on outcomes. The relatively lower accuracy of risk prediction using family history compared with AI/PRS means that more true high-risk women, who are incorrectly predicted as low risk based on family history, benefit from biennial screening. If these benefits outweigh the disutility from more frequent screening for low-risk women, effectiveness of ‘Family history + biennial screening for low risk’ can still be higher than ‘Family history + no screening for low risk’.

Meanwhile, under ‘Annual screening for all’, all high-risk women are correctly classified. In addition, some low-risk women who still develop cancer also benefit from annual screening. As a result, fewer cases are missed compared with ‘AI/PRS + biennial screening for low risk’. Even though ‘Annual screening for all’ also carries the burden of more frequent screening for low-risk women, the total effectiveness can still be higher than ‘AI/PRS + biennial screening for low risk’ if the utility gains from fewer missed cases more than offset the disutility from more frequent screening.

Our study provides useful insights to inform the ongoing debate over appropriate breast cancer screening practices for women aged between 40 and 49 years. We find that using AI to risk-stratify women and targeting screening at only high-risk women can generate greater economic value than existing screening guidelines. Compared with family history-based screening (which reflects current USPSTF guidelines), this AI-based strategy can help alleviate existing concerns about delayed diagnoses as more high-risk women would be accurately identified and screened. At the same time, it can reduce false-positive diagnoses from screening all women over age 40 annually (as recommended by ACOG/ACR guidelines).

Our study has several limitations. First, randomized controlled trials that directly compare AI with PRS or existing screening criteria are lacking. Thus, data on efficacy of AI and PRS had to be obtained from different studies and demographic and personal risk factors considered in addition to AI and PRS differed slightly in the two studies. Second, cost of using AI for breast cancer risk prediction in clinical practice is not yet known and was not available from existing literature. Therefore, for our analysis, we had to rely on cost estimates from the European Society of Radiology [21] to estimate this cost. Nevertheless, we varied the cost of AI in one-way sensitivity analyses and our results continued to hold for all costs of AI as high as $318 per mammogram. Finally, in our model, AI was used to guide breast cancer screening over a 10-year duration (i.e., between ages 40 and 49) while existing data could validate the accuracy of AI-based risk prediction only for 5 years post risk-assessment [7]. However, these existing data provide suggestive evidence that AI is able to detect features associated with long-term risk [7]. As deep learning models improve in the future and long-term data become available, future studies could re-examine the cost-effectiveness of using AI to guide breast cancer screening not just among women aged 40–49 but in women across the entire candidate age range, including those over age 50.

Despite these limitations, our study can serve as a useful starting point to stimulate and inform future research and policy choices about breast cancer screening guided by novel AI technologies. Furthermore, it provides a general framework that can be easily updated (when new data on AI risk prediction become available) or adapted to study cost-effectiveness of using AI in other disease domains.

## Conclusions

This study finds that using AI to risk-stratify women for breast cancer screening between ages 40 and 49 (followed by screening based on existing guidelines beyond age 50) is cost-effective compared with screening based on PRS or family history, annual screening for all women and no screening. By accurately identifying and screening more high-risk women and avoiding screening for low-risk women, this cost-effective AI-based screening strategy can help address existing concerns about delayed diagnoses as well as false-positive diagnoses that could arise with conventional screening strategies.

## Supplementary Information

**Additional file 1.**

## Data Availability

Data used in this analysis were obtained from the published literature and references to sources have been provided in the manuscript. For further information, please contact the corresponding author Dr. Hai Nguyen at hvnguyen@mun.ca.

## Abbreviations

AI: Artificial Intelligence
PRS: Polygenic Risk Score
QALY: Quality Adjusted Life Year
USPSTF: US Preventive Services Task Force
ACS: American Cancer Society
AUC: Area under the Receiver Operating Characteristic Curve
ICER: Incremental Cost-Effectiveness Ratio
WTP: Willingness to Pay
PSA: Probabilistic Sensitivity Analysis
ER: Estrogen Receptor
HER2: Human Epidermal Growth Factor
ACOG: American College of Obstetricians and Gynecologists
ACR: American College of Radiology
