# Supplementary material for: Cost-effectiveness of using artificial intelligence versus polygenic risk score to guide breast cancer screening
Source: BMC Cancer. 2022 May 6;22:501. doi: 10.1186/s12885-022-09613-1 (PMC9074290; doi:10.1186/s12885-022-09613-1)
Supplement: Supplementary file 1 — Additional file 1. [file 12885_2022_9613_MOESM1_ESM.pdf]

ONLINE SUPPLEMENTARY MATERIALS

S.1 Estimation of Model Inputs

Incidence of breast cancer: Incidence rates of in-situ breast cancer were obtained from age-specific incidence rates reported by the Surveillance, Epidemiology, and End Results (SEER) program for white women for 2013-2017 [1]. To account for the increase in incidence rates due to mammography screening, we applied relative risk adjustments similar to Schousboe et al. Following their approach, age specific incidence of in-situ cancer was 0.26 times the rates for 2013-2017 for white women who were not screened and 1.02 times the rates for 2013-2017 for those who were screened.

Incidence rates of invasive breast cancer were also taken from age-specific incidence rates reported by the SEER program for white women for 2013-2017 [1]. Incidence of invasive cancer from 'No tumor' and 'In situ' cancer states was then calculated following the method described by Schousboe et al. [2].

Breast cancer mortality: Patients diagnosed with invasive breast cancer faced risk of breast cancer mortality for up to 20 years after diagnosis. This risk was specific to age and stage at diagnosis as well as estrogen-receptor (ER) and human epidermal growth factor 2 (HER2) status and was calculated from survival estimates reported by Munoz et al. [3]. Munoz et al. estimated patient survival in the absence of adjuvant treatment. To capture the mortality hazard reductions from adjuvant treatment, we applied hazard reduction of 0.67 from treatment with adjuvant hormonal therapies (such as tamoxifen) for patients with positive hormone receptor status, and 0.66 from treatment with trastuzumab for those with positive HER2 status [4].

S.2 Augmented Base Case Analysis

Table A1: Lifetime Costs, QALYs and breast cancer outcomes by Screening Strategy, Augmented Base Case Analysis

Strategy	Cost (in 1000 $)	Effectiveness (in QALYs)	ICER ($/QALY)
No screening	1,696,973	1,976,505	--
Family history + no screening for low risk	1,799,474	1,978,189	Extended dominated
PRS + no screening for low risk	1,806,935	1,980,544	Dominated
AI + no screening for low risk	1,808,432	1,981,085	24,335
PRS only + no screening for low risk	1,838,698	1,980,473	Dominated
AI only + no screening for low risk	1,840,129	1,980,658	Dominated
Family history + biennial screening for low risk	1,853,902	1,980,796	Dominated
PRS + biennial screening for low risk	1,882,724	1,978,519	Dominated
AI + biennial screening for low risk	1,890,769	1,978,761	Dominated
PRS only + biennial screening for low risk	1,909,832	1,978,495	Dominated
AI only + biennial screening for low risk	1,915,226	1,978,406	Dominated
Annual screening for all	1,990,750	1,978,491	Dominated

Note: All costs are in 2020 US dollars ($). Costs and effectiveness are calculated per 100,000 women. All strategies (except 'No screening') involve annual screening for women identified as high-risk. 'AI' refers to risk prediction accounting for both AI and other risk factors. 'PRS' refers to risk prediction accounting for both PRS and other risk factors. 'AI only' refers to risk prediction accounting for only AI without other risk factors. 'PRS only' refers to risk prediction accounting for only PRS without other risk factors. Beyond age 50, women without family history are screened biennially and those with family history are screened annually in all strategies except 'No screening' and 'Annual screening for all' strategies.

References:

1. National Cancer Institute Surveillance, Epidemiology, and End Results Program. Breast Cancer SEER Incidence Rates by Age at Diagnosis, 2013-2017. 2020. https://seer.cancer.gov/explorer/application.html.

2. Schousboe JT, Kerlikowske K, Loh A, Cummings SR. Personalizing mammography by breast density and other risk factors for breast cancer: analysis of health benefits and cost-effectiveness. Annals of internal medicine. 2011;155:10–20.

3. Munoz DF, Plevritis SK. Estimating breast cancer survival by molecular subtype in the absence of screening and adjuvant treatment. Medical Decision Making. 2018;38 1_suppl:32S-43S.

4. Shih Y-CT, Dong W, Xu Y, Shen Y. Assessing the cost-effectiveness of updated breast cancer screening guidelines for average-risk women. Value in Health. 2019;22:185–93.
